# Lysophosphatidic Acid and Glutamatergic Transmission

**DOI:** 10.3389/fnmol.2019.00138

**Published:** 2019-05-28

**Authors:** Carolina Roza, José A. Campos-Sandoval, María C. Gómez-García, Ana Peñalver, Javier Márquez

**Affiliations:** ^1^Departamento de Biología de Sistemas, Edificio de Medicina Universidad de Alcalá, Alcalá de Henares, Spain; ^2^Laboratorio de Química de Proteínas, Departamento de Biología Molecular y Bioquímica, Facultad de Ciencias, Instituto de Investigación Biomédica de Málaga (IBIMA), Universidad de Málaga, Campus de Teatinos, Málaga, Spain

**Keywords:** LPA, glutamatergic transmission, synaptic plasticity, glutaminases, neuropathic pain

## Abstract

Signaling through bioactive lipids regulates nervous system development and functions. Lysophosphatidic acid (LPA), a membrane-derived lipid mediator particularly enriched in brain, is able to induce many responses in neurons and glial cells by affecting key processes like synaptic plasticity, neurogenesis, differentiation and proliferation. Early studies noted sustained elevations of neuronal intracellular calcium, a primary response to LPA exposure, suggesting functional modifications of NMDA and AMPA glutamate receptors. However, the crosstalk between LPA signaling and glutamatergic transmission has only recently been shown. For example, stimulation of presynaptic LPA receptors in hippocampal neurons regulates glutamate release from the presynaptic terminal, and excess of LPA induce seizures. Further evidence indicating a role of LPA in the modulation of neuronal transmission has been inferred from animal models with deficits on LPA receptors, mainly LPA_1_ which is the most prevalent receptor in human and mouse brain tissue. LPA_1_ null-mice exhibit cognitive and attention deficits characteristic of schizophrenia which are related with altered glutamatergic transmission and reduced neuropathic pain. Furthermore, silencing of LPA_1_ receptor in mice induced a severe down-regulation of the main glutaminase isoform (GLS) in cerebral cortex and hippocampus, along with a parallel sharp decrease on active matrix-metalloproteinase 9. The downregulation of both enzymes correlated with an altered morphology of glutamatergic pyramidal cells dendritic spines towards a less mature phenotype, indicating important implications of LPA in synaptic excitatory plasticity which may contribute to the cognitive and memory deficits shown by LPA_1_-deficient mice. In this review, we present an updated account of current evidence pointing to important implications of LPA in the modulation of synaptic excitatory transmission.

## Introduction

**Figure 1:**
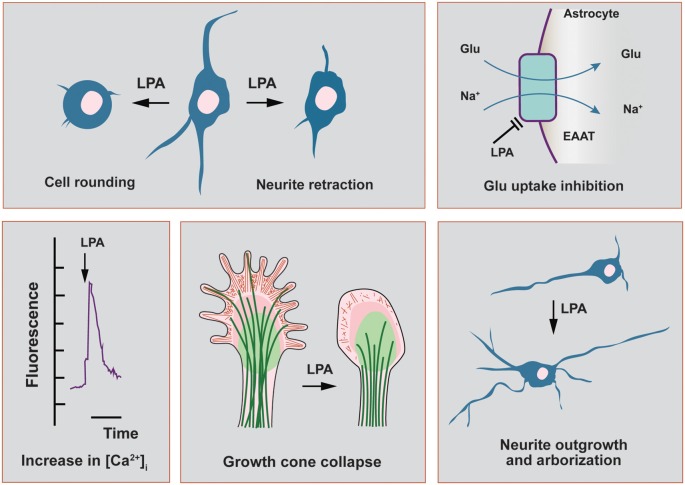
Main functional effects of lysophosphatidic acid (LPA) in cells related to the nervous system. In this scheme, we highlight the main functional effects elicited by LPA in neuronal cell lines and in primary cultures of neurons and astrocytes. Neurite retraction, growth cone collapse and cell rounding have been described to be mediated by all LPA receptor subtypes, except LPA_3_. This last receptor has been recently shown to induce neurite branch formation. In neurons, LPA also induces a sustained elevation of intracellular calcium, with strong repercussions on cytoskeletal remodeling as neurite outgrowth. The morphological changes induced by LPA in neurons can be also mediated by autocrine effects coming from neighbor astrocytes, which promote neuronal differentiation and increases in arborization and neurite outgrowth. Finally, LPA-treated astrocytes show a range of different effects including the inhibition of glutamate uptake and increases in intracellular calcium concentration.

Phosphatidic acid (PA) or phosphatidate (diacylglycerol 3-phosphate) is the simplest phosphoglyceride and the precursor of important glycerophospholipids. Only small amounts of PA are present in eukaryotic membranes; however, the molecule is a key intermediate in the biosynthesis of the other phosphoglycerides. Lipids are one of the most abundant biomolecules in mammalian brain; in fact, they outnumber the amount of proteins and other cellular components in human brain accounting for 60%–70% of total dry weight (Svennerholm et al., 1994). Apart from their structural role forming the lipid bilayer of cell membranes, where proteins are embedded, many lipids also possess key cell signaling functions and the term “bioactive lipids” has been coined to refer to those non-structural regulatory functions. Lysophosphatidic acid (LPA; 1 or 2-acyl-sn-glycero-3-phosphate) is a naturally occurring lysophospholipid derived from cell membranes that belong to the growing list of bioactive lipids. It may act as an intercellular signaling molecule after recognition by G protein-coupled receptors (GPCRs; Ye et al., 2002; Fukushima, 2004; Choi and Chun, 2013). LPA is a heterogeneous molecule, with distinct variants regarding the length and degree of saturation of its acyl chains (Moolenaar and Hla, 2012), which can be recognized by at least six cognate GPCRs (named LPA_1_ to LPA_6_) present in many different cell types (Mutoh et al., 2012); hence, a plethora of biological responses have been described for this phospholipid messenger, including differentiation, proliferation, migration, vascular regulation, cell survival and cytoskeletal remodeling (Choi and Chun, 2013; Figure 1). In this review article, we will focus on LPA actions in synaptic transmission, plasticity and neuropathic pain. We will present a succinct account of experimental results dealing with primary cell cultures of neurons and astrocytes, neuronal cell lines as well as knock-out (KO) mouse models of different LPA receptors with special emphasis on KOLPA_1_ mice.

## LPA and Synaptic Plasticity

Glutamate, the main excitatory neurotransmitter in the nervous system, activates both ionotropic (NMDAR and AMPAR) and metabotropic receptors. NMDAR is composed of heteromultimeric subunits and display slow channel kinetics and a marked permeability to Ca^2+^. However, at rest, they are blocked by Mg^2+^ and only upon neuronal depolarization they become fully functional. On the other hand, AMPAR isoforms display lower voltage-dependence and faster kinetics than NMDARs and permeate predominantly Na^+^. Furthermore, AMPARs display a lower affinity for glutamate and rapidly desensitize upon their selective stimulation. In neurons, AMPARs undergo constitutive and activity dependent-trafficking which is relevant for synaptic plasticity—reviewed in Lüscher and Malenka (2012).

The first direct evidence showing that LPA had a modulatory role on glutamate receptors came from early studies in Xenopus oocytes and isolated hippocampal neurons, where glutamate- and NMDA-mediated currents were potentiated by direct application of LPA (Tabuchi et al., 1997; Lu et al., 1999). This effect was indirectly mediated by protein kinase C (PKC)-dependent phosphorylation of the NMDAR (Lu et al., 1999) upon extracellular Ca^2+^ entering the cell *via* this channel type (Holtsberg et al., 1997). Since then, a series of experimental evidence have shown that LPA acts as a fine-tuning regulatory mechanism of glutamatergic transmission in the nervous system.

NMDA-mediated Ca^2+^ increases in the postsynaptic neuron are able to induce long-term forms of either potentiation (LTP) or depression (LTD) at both excitatory and inhibitory synapses (Bliss and Schoepfer, 2004). LTP and LTD represent well established experimental correlates of learning and memory (Bliss and Collingridge, 1993). Since LPA_1_-null mice showed impairments in spatial learning and memory (Santin et al., 2009; Castilla-Ortega et al., 2010), it is reasonable to assume a modulatory role of LPA_1_ in these processes. Despite the cellular and molecular mechanisms are still poorly understood, the described behavioral alterations could be explained by an abnormal development of certain brain areas (further discussion in “LPA and Structural Plasticity” section) but also by a reduced signaling *via* NMDAR (Blanco et al., 2012; Shin et al., 2012). Chronic exposure to cocaine and other drugs of abuse induce an abnormal increase in basal locomotion—i.e., conditioned locomotion—which correlates with the strengthening of glutamatergic synapses *via* LTP mechanisms (Kauer and Malenka, 2007) but also with a reduction in the density of dendritic spines (Scofield et al., 2016). LPA_1_-null mice exhibited reduced cocaine-induced conditioned locomotion, which was accompanied by a reduction of metabotropic mGluR3 receptor (Blanco et al., 2012). Therefore, it is plausible to assume a role of LPA in conditioned locomotion both through NMDAR mediated synapse, but also by means of morphological changes (further discussed in next section). Ginseng (the root of *Panax ginseng*), widely used in traditional medicine, contains gintonin, a unique LPA-ginseng protein complex which can deliver LPA to is cognate receptors with high affinity (Hwang et al., 2012b; Choi et al., 2015). It has been shown that application of gintonin enhanced NMDA current in oocytes, induced transient elevations of intracellular Ca^2+^ in cultured hippocampal neurons and favored LTP in rat hippocampal slices (Shin et al., 2012). Besides, long-term oral administration of this compound was able to attenuate memory impairments in a transgenic mouse model of Alzheimer’s disease (Hwang et al., 2012a; Choi et al., 2015).

**Figure 2:**
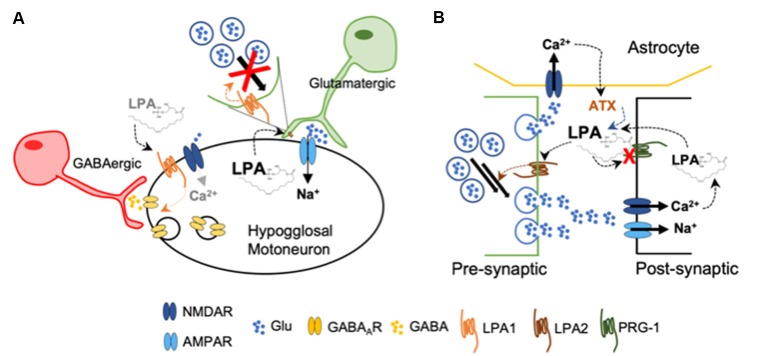
**(A)** Upon high levels of LPA, glutamatergic pre-synaptic neurons decrease the pool of available glutamate-containing vesicles acting as a negative feed-back to decrease excitability of hypoglossal motoneurons. Low concentration of LPA induces gamma-aminobutyric acid receptor type A (GABA_A_R) internalization at the post-synaptic membrane, increasing briefly the excitability of the hypoglossal motoneuron by disinhibition (García-Morales et al., 2015). **(B)** Loss of functional plasticity-related gene 1 (PRG-1) at the post-synaptic membrane leads to increased LPA accumulation at the synaptic cleft. Activation of LPA_2_ facilitates now glutamate release from the presynaptic neuron. In addition, the release of autotaxin (ATX) from astrocytes is now further increased by the enhanced concentration of glutamate (Trimbuch et al., 2009; Vogt et al., 2016; Thalman et al., 2018).

Direct evidence on the regulatory effects of LPA in synaptic strength comes from *in vitro* studies in hypoglossal motoneurons expressing AMPAR and gamma-aminobutyric acid receptor type A (GABA_A_R). During inspiration, these motoneurons are synaptically excited by glutamatergic brain stem afferents and activate tongue muscles to maintain air inflow towards the lungs. They present a pattern of rhythmic firing in bursts that follows the respiratory phases (Peever et al., 2002). Studies using brain stem slices obtained from neonatal mice, demonstrated that LPA, *via* LPA_1_, induced a rapid and reversible depression on synaptic strength (García-Morales et al., 2015), which is another form of functional synaptic plasticity (Schneggenburger et al., 2002). The authors demonstrated that LPA signaling on excitatory glutamatergic transmission takes place at the presynaptic neuron, as the attenuation of excitatory AMPA-current by LPA was related to a reduction in glutamate release probability. The LPA-induced mechanism is mediated by a phospholipase C (PLC)-dependent phosphorylation of the myosin light chain (MLC) by MLC kinase at the presynaptic neuron (García-Morales et al., 2015). Thus, the pool-size of available synaptic vesicles within excitatory boutons is reduced. Such depletion of available vesicles usually underlies short-term forms of synaptic depression (Schneggenburger et al., 2002). By this means, LPA released from the postsynaptic neuron acts as a retrograde messenger by reducing the strength of the synapse and, hence, acting as a negative-feedback mechanism preventing hyperactivity (Figure 2A). In the context of synaptic plasticity, it has already been proposed the synthesis of a retrograde messenger that diffuses to the presynaptic ending, whereby interacts with specific targets and regulates neurotransmitter release (Regehr et al., 2009). On the other hand, at GABAergic synapses, LPA exerts a post-synaptic effect by favoring receptor internalization, leading to neuronal disinhibition. This mechanism is mediated by dephosphorylation of the GABA_A__γ2_ subunit upon recruitment of RhoA/ROCK-calcineurin (García-Morales et al., 2015) and resembles typical LTD (Bliss and Schoepfer, 2004). Modest opening of NMDARs—i.e., less Ca^2+^ entry—leads to the activation of calcineurin—calcium/calmodulin-dependent protein phosphatase due to its higher affinity for calcium/calmodulin than CaMKII (Bliss and Schoepfer, 2004). Experiments performed *in vivo* showed that blockade of LPA signaling by LPA_1–3_ antagonists increased the frequency of the spikes fired per burst. This demonstrated that endogenous LPA signaling contributes to normal patterns of motor output commands in adult mice (García-Morales et al., 2015) and could help to explain the impairment in suckling behavior—regulated by hypoglossal neurons—described in LPA_1_-null mice (Contos et al., 2000). The apparent mismatch of LPA effects in hypoglossal neurons might be related with activity-dependent LPA synthesis and/or release, promoting neuronal disinhibition (increased firing) at low concentrations and depressing excitation (reduced firing) upon increased activity.

Further experimental data obtained from different research groups also suggest a facilitator role of LPA during glutamatergic transmission. Plasticity-related gene 1 (PRG-1) is a specific membrane protein present at the postsynaptic spines of glutamatergic synapses in the brain cortex and hippocampus, where it has a key role modulating excitability (Vogt et al., 2016). PRG-1 is involved in LPA uptake into intracellular postsynaptic compartments (Trimbuch et al., 2009), thereby controlling LPA levels in the synaptic cleft. When PRG-1 is absent, mice develop severe seizures; however, double PRG-1/LPA_2_ null mice do not exhibit an epileptic phenotype. When PRG-1 function is restored, the excitability levels examined *in vitro* in individual cells returned to normal. Interestingly, when the LPA-PRG-1 interacting site was altered, hyperexcitability remained (Trimbuch et al., 2009). Later on, a mutation of the *Prg-1* gene was found which blocks its ability to regulate cellular LPA uptake, leading to an increased hyperexcitability (Vogt et al., 2016). In PRG-1^+/−^ mice, inhibition of the LPA-synthesizing enzyme autotaxin (ATX) normalized cortical hyperexcitability and behavior (Thalman et al., 2018). The expression of ATX was restricted to the astrocytes located in excitatory synapses, while its secretion to the synaptic cleft was regulated by glutamatergic signaling (Thalman et al., 2018; Figure 2B).

Both PRG-1^+/−^ and LPA_1_ null-mice display deficits in prepulse inhibition (PPI) during the startle reflex in comparison with their respective wild-types (WTs; Harrison et al., 2003; Thalman et al., 2018). PPI is considered as a useful tool to study the neurobiology of schizophrenia, although it is also a common phenotype of other neuropsychiatric disorders (Swerdlow et al., 2008). The exact mechanisms underlying schizophrenia are poorly understood and beyond the scope of this review, but it has been proposed that the dysregulation of glutamatergic neurotransmission is a key component in this pathophysiology (Moghaddam and Javitt, 2012; McNally and McCarley, 2016). Increasing evidence points to an enhanced glutamatergic transmission through activation of AMPAR due to the presence of defective NMDAR (Hardingham and Do, 2016; Moghaddam and Javitt, 2012). But also LPA_1_-null mice show a reduction in the generation of gamma-osciliations (Cunningham et al., 2006). This type of rhythmic activity at 30–100 Hz allows coherent synchronous communication between different brain areas to evoke adequate perception. It has been postulated that a reduction of NMDAR activity in a subset of GABAergic interneurons results in a disruption of the synchrony, leading to an “integration disorder” (McNally and McCarley, 2016). Abnormal gamma-band activity frequently observed in patients with schizophrenia is strongly correlated with perceptual impairments (McNally and McCarley, 2016). In this line, ketamine (a non-competitive NMDAR antagonist) superfusion in entorhinal slices from WT littermates of LPA_1_-null mice was able to reduce gamma-oscillations (Cunningham et al., 2006). Taken together, the results point to a reduction in glutamatergic transmission *via* NMDAR, at least in some brain areas, but the exact mechanisms by which glutamate release is altered or which NMDAR is malfunctioning are still unknown.

A deficit in LPA-LPA_1_ leading to a reduction in NMDAR transmission could be explained by experimental data obtained from LPA_1_-null mice: low hippocampal levels of both glutamine and GABA (Harrison et al., 2003; Roberts et al., 2005), a reduced release of glutamate at the hippocampus (Roberts et al., 2005) or a decreased hippocampal synaptic activity (Musazzi et al., 2011). However, Harrison and coworkers did not observe changes in synaptic function at the hippocampus (Harrison et al., 2003) while an enhanced level of SNARE proteins were described at the presynaptic site (Musazzi et al., 2011), which would point to a favored neurotransmitter release. On the other hand, and in apparent contradiction, a lack of functional PRG points to an excess of LPA as a contributing factor during the development of mental disorders present with PPI deficits (Vogt et al., 2016). Thus inhibition of LPA synthesis reduced excess of glutamate release and restored PPI in PRG^+/−^ mice (Vogt et al., 2016).

Proper brain functioning requires an adequate balance between excitatory and inhibitory inputs. Despite the mechanisms of actions are not fully understood, LPA regulates glutamatergic transmission in such a way that a disruption in these signaling pathways seems to have an impact in the development of cognitive disorders.

## LPA and Structural Plasticity

The role of membrane-derived phospholipids as key regulators of synaptic neurotransmission and plasticity was early detected in pioneer works measuring responses of different cell types to these biomediators (reviewed in Moolenaar, 1995; Bieberich, 2012). To discuss the effects of LPA on structural plasticity we should take into account that LPA targets many brain cells, including those forming the tripartite synapse (presynaptic and postsynaptic neurons and astrocytes), as well as neural progenitor cells (NPCs), microglia, oligodendrocytes, Schwan cells and endothelial cells (Noguchi et al., 2009). Thus, LPA was shown to induce a wide range of responses in the central nervous system (CNS) as well as in neural cell lines (Figure 1). LPA elicited sharp changes in cell’s shape and morphology, growth cone collapse and neurite retraction in many neuronal cell lines through activation of the small GTPase Rho which in turn contracts cytoskeleton (Ye et al., 2002; Choi et al., 2010; Fukushima et al., 2011). Several Rho-mediated intracellular signals seem to be involved in the cytoskeletal rearrangements of neuronal cells after activation of protein G13 by LPA binding (reviewed in Fukushima, 2004). The activation of Rho kinase produces phosphorylation of the MLC and inactivation of myosin phosphatase, which triggers actin polymerization and cell contraction (neurite retraction; Figure 1). The LPA-dependent Rho kinase-mediated regulation of neurite outgrowth can be strongly influenced by the intracellular Ca^2+^ levels, as was recently shown in primary hippocampal neurons cultured with agonists and antagonists of Rho kinase and Ca^2+^ (Ji et al., 2017).

LPA strongly influences the intracellular Ca^2+^ levels and thus may play important roles in a variety of functions as cellular shape regulation, motility and apoptosis (Figure 1). Neuronal calcium levels are modulated by many proteins, including ligand- and voltage-gated channels, pumps, transporters and calcium-binding proteins, and play a crucial role in neuronal development, adaptive responses, dendritic spine development and synaptic plasticity, as well as in pathological neuronal processes (Segal and Korkotian, 2016; Jackman and Regehr, 2017). In neurons, LPA induces a sustained elevation of intracellular calcium which has been related to extracellular glutamate levels and modulation of glutamate receptors (Ye et al., 2002), as discussed in the previous section. However, pathophysiological LPA concentrations in brain, as happens after hemorrhagic brain injury when a dramatic increase in LPA concentration is observed in CSF, could lead to persistent Ca^2+^ increases conducive to neuronal necrosis and apoptosis (Holtsberg et al., 1997; Steiner et al., 2002).

The processes of neuronal arborization and neurite outgrowth are strongly influenced by LPA, which has been described as an important regulator of morphological neuroplasticity (Shiono et al., 1993; Figure 1). After being recognized by LPA receptors, cyclic phosphatidic acid (cPA) may also elicit a neurotrophic effect and promotes neurite outgrowth in embryonic hippocampal neurons acting through the same effectors as nerve growth factor (NGF), including a sustained activation of ERK 1/2 and Akt (Fujiwara et al., 2003). Of interest, a novel signaling pathway involving LPA_3_ receptor, Gq protein and the Rho family GTPase 2 (Rnd2) has been lately reported to play an important role in neurite branching of hippocampal neurons and, therefore, on neural network formation (Furuta et al., 2012). In sharp contrasts with the reported effects of LPA on neurite retraction in many neuronal cell types, which were mostly mediated by all LPA receptors except LPA_3_ (Ishii et al., 2000; Fukushima, 2004), these authors demonstrated a novel signaling pathway linking LPA_3_ with enhanced axonal branch formation, both in neuronal cell lines with ectopic expression of LPA_3_ as well as in primary neurons with endogenous LPA_3_ expression (peak of expression on postnatal day 7; Furuta et al., 2012).

**Figure 3:**
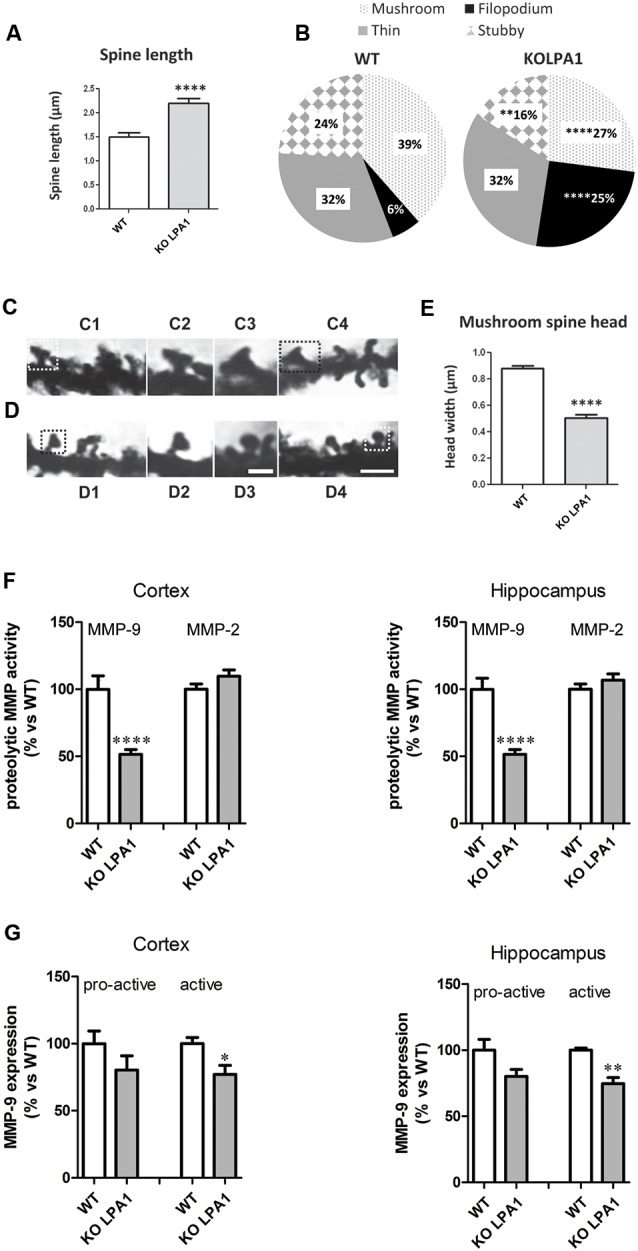
Golgi staining of CA1 pyramidal neurons from wild-type (WT) and KOLPA_1_ mice demonstrating the morphological differences between KOLPA_1_ and WT spines.** (A)** Overall spine length values showed significant differences between genotypes (WT 1.4 ± 0.05 μm, KOLPA_1_ 2.12 ± 0.09 μm; Mann-Whitney, ****p < 0.0001). **(B)** Significant differences were found in the distribution of dendritic spine morphologies within stratum oriens from KOLPA_1_ model compared to the same region of WT mice. Filopodia type was significantly more frequent in KOLPA_1_ mice in detriment of both mushroom and stubby categories (*t*-test, Filopodia ****p < 0.0001, Stubby **p = 0.0073 and Thin, n.s.; Mann-Whitney, Mushroom ****p < 0.0001). **(C,D)** Spine head of mushroom subtype was smaller in KOLPA_1_ (**D**; D2, D3 depict details from D1 and D4, respectively) than in WT mice (**C**; C2, C3 depict details from C1 and C4, respectively). **(E)** Quantitative analysis demonstrated a significant decrease in the head-width of mushroom spines from KOLPA_1_ mice (0.52 ± 0.02 μm) compared to WT (0.91 ± 0.02 μm); *t*-test, ****p < 0.0001. Scale bar: C1, C4, D1 and D4 (2 μm); C2, C3, D2, D3 (1 μm). Matrix metalloproteinase (MMP) activities **(F)** and protein expression levels **(G)** in cerebral cortex and hippocampus of WT and KOLPA_1_ mice. Significant differences were found in MMP-9 proteolytic activity of both brain areas **(F)**, showing a decreased activity in knock-out (KO) mice (****p < 0.0001), while MMP-2 remained unchanged. Immunoblot analysis of MMP-9 protein expression in cerebral cortex and hippocampus from WT and KOLPA_1_ mice **(G)**. Quantitive analysis of proactive (92 kDa) and active (82 kDa) MMP-9 were determined by densitometry and relative to β-actin expression (WT *n* = 4; KO *n* = 5). A reduction in active MMP-9 expression was detected in KOLPA_1_ compared to WT mice (*p < 0.05, **p < 0.01), while no changes were detected in pro-active MMP-9 expression (Adapted from Peñalver et al., 2017).

Sprouting, extension and arborization of neurites, formation of novel dendrites and remodeling (maturation) of existing ones are key structural changes with strong implications in synaptic formation and plasticity. In this context, modulation of dendritic spine dynamics by LPA has been previously reported; for instance, LPA_1_ was localized in dendritic spines and its overexpression in cultured hippocampal neurons correlated with morphological and functional effects: increases in the density and size of spines and significant changes in the decay time of miniature excitatory synaptic currents (mEPSC; Pilpel and Segal, 2006). Of note, none of the three classic pathways activated by LPA (Gq-mediated activation of PKC, Gi inhibition of adenyl cyclase and G12/13 activation of Rho) mediates the effects on spines of LPA_1_ overexpression (Pilpel and Segal, 2006). In agreement with these results, changes in the morphology of excitatory synaptic spines, with potential repercussion in synaptic neurotransmission, were found after genetic deletion of LPA_1_ receptor in a murine model; namely, hippocampal dendritic spines showed a clear immature phenotype as compared with WT counterparts, with predominance of filopodia in detriment of the mushroom and stubby spines, and marked reduction in mushroom spine head-width (Figure 3; Peñalver et al., 2017).

LPA-induced effects on neuronal network formation are multiple and highly depending on the physiological and developmental states. Moreover, the effects of LPA on neuronal differentiation could be also mediated in an indirect way through previous stimulation of surrounding astrocytes. In fact, LPA induces a wide range of responses in cultured astrocytes, including proliferation and inhibition of glutamate uptake (Steiner et al., 2002; Shano et al., 2008; Figure 1), inhibition of glucose uptake, stimulation of lipid peroxidation and increases in intracellular calcium concentration (Keller et al., 1997). Interestingly, LPA-primed astrocytes promote neuronal differentiation of cortical cerebral progenitors and developing neurons, which showed increases in arborization and neurite outgrowth (de Sampaio e Spohr et al., 2008, 2011). These effects were exclusively ascribed to astrocytic LPA_1_ and/or LPA_2_ receptors, but not LPA_3_ (de Sampaio e Spohr et al., 2008). Furthermore, the contribution of LPA-activated astrocytes to neuronal differentiation is also supported by data on expression levels of LPA receptors during mouse brain development. Thus, LPA_1_ receptor was found in astrocytes and oligodendrocytes, as well as in neurons, during embryonic and postnatal development (Suckau et al., 2019). However, LPA_1_ protein was not detectable in mature hippocampal neurons (14-day-old cultures), when an extensive network of synaptic connections was established, but was strongly detected in glial cells and immature neurons (only 2 days in culture; Suckau et al., 2019). In addition, the production of NGF is also enhanced by astrocytes’ exposure to LPA, which may further contribute to neuronal differentiation (Furukawa et al., 2007).

On the other hand, LPA signaling *via* LPA_1_ receptor has been associated with invasive and metastatic properties of cancer cells, particularly through the induction of proteolytic enzymes like matrix metalloproteinases (MMPs) 2 (Kato et al., 2012) and 9 (Park et al., 2011). Interestingly, we also found a link between LPA_1_ and MMP-9 expression in brain: silencing LPA_1_ expression in mouse brain induced a marked reduction in MMP-9 activity in hippocampus and cortex of KOLPA_1_ mice compared with WT counterparts, while no changes were detected for MMP-2 (Figure 3; Peñalver et al., 2017). A considerable body of evidence has recently shown a key role for MMP-9 in synaptic remodeling, synaptic plasticity and cognitive processes (Rivera et al., 2010; Michaluk et al., 2011). In neurons, MMP-9 is located at the postsynaptic domains of excitatory synapses and its inhibition has been linked to memory deficits in behavioral learning paradigms (Nagy et al., 2007). MMP-9 is a key regulator of dendritic spine morphology (Michaluk et al., 2011; Dziembowska and Wlodarczyk, 2012); thus, its activation through proteolytic cleavage of an inactive precursor form (pro-active MMP-9) is strictly regulated during spine maturation (Tian et al., 2007; Bilousova et al., 2009). Immunoblot analysis indicates that active MMP-9 protein levels in brain are reduced in KOLPA_1_ respect to WT mice, although no significant differences were found for pro-active MMP-9 (Figure 3). The downregulation of MMP-9 activity shown by LPA_1_-null mice may play an important role in their cognitive performance and partially explain the neurophysiological phenotype shown by these animals (Santin et al., 2009; Castilla-Ortega et al., 2010). These results suggest that a reduced MMP-9 activity would be a downstream effect of the absence of LPA-induced Rho/ROCK signaling pathway, which is known to produce proteolytic enzymes like MMP-9 (Yu et al., 2014).

**Figure 4:**
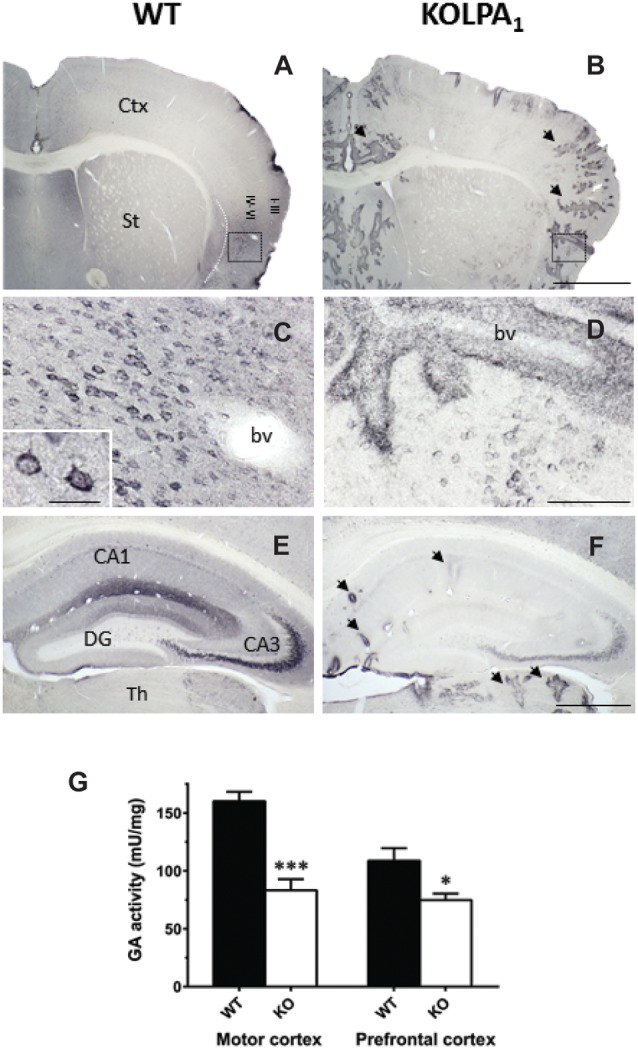
Comparative analysis of immunostaining for *Gls*-encoded long GA protein variant (KGA) in WT and KOLPA_1_ mice brain. Specific immunolabeling for KGA was detected within the somata/neuropile of several brain regions in WT brain (left column). In general, there was a strong reduction in the staining together with a progressive switch to a perivascular location from control to KO mice. **(A,B)** Panoramic views of sections containing cerebral cortex and striatum: black arrows indicate KGA-positive blood vessels. **(C,D)** Progressive change of KGA-staining in deep layers of agranular insular cortex (square; detail of cortical somatic staining, inset in **(C)**. **(E,F)** KGA-positive staining in the CA1-CA3 hippocampal subfields and DG was dramatically decreased in KO genotype in comparison to WT. Ctx, cortex; St, striatum; bv, blood vessel; CA1, CA3, hippocampal subfields; DG, dentate gyrus; Th, thalamus. Scale bar: **(A,B)**, 1 mm; **(C,D)**, 100 μm; **(E,F)**, 500 μm; Inset **(C)**, 25 μm. Determination of total GA activity in mouse brain regions** (G)**. Significant differences in GA specific activity were found in motor cortex (WT *n* = 9; KO *n* = 5) and prefrontal cortex (PFC; WT *n* = 12; KO *n* = 8), but not in hippocampus and striatum (results not shown). ***p < 0.001; *p < 0.05 (both panels adapted from Peñalver et al., 2017).

A further relationship between LPA signaling and glutamatergic excitatory transmission was recently found in LPA_1_-null mice. In this model, a marked down-regulation of the main glutaminase (GA) isoform (KGA) involved in the synthesis of neurotransmitter glutamate was reported (Peñalver et al., 2017). The drastic decrease of KGA protein expression was particularly relevant in cerebral cortex and hippocampus (Figure 4), although correlation between GLS mRNA abundance, KGA protein levels and GA activity was only apparent in prefrontal cortex (PFC) and motor cortex (Figure 4). Despite substantial decrements of KGA protein in key glutamatergic regions, the total GA activity was essentially maintained which immediately suggests activation of compensatory mechanisms. For example, two distinct LPA-related mechanisms, involving other LPA receptors, would compensate this regional KGA loss by enhancing the enzymatic activity of GLS isoforms. The *Gls*-encoded alternative spliced GAC isoform is activated by Rho GTPases (Wang et al., 2008); interestingly, LPA stimulates activation of Rho in several neuronal cell lines, cortical progenitors and cortical neurons (Ye et al., 2002). Moreover, the Raf-Mek-Erk signaling pathway has been shown to increase KGA activity by phosphorylation after stimulation by several mitogens, including LPA (Thangavelu et al., 2012); therefore, post-translational modifications of the remaining KGA protein pool would cooperate in keeping basal GA activity in mutant KOLPA_1_ mice.

The deficit of KGA protein in null mutant mice lacking LPA_1_ receptors may also give rise to developmental defects in cortical and hippocampal neurons with strong implications in synaptic plasticity. Of note, in null mice for the *Gls* gene the main functional deficit was found in the implementation of more active neural network circuits, stressing the importance of KGA in neuronal maturation, development and differentiation (Masson et al., 2006). In accordance with these findings, GLS isoforms (KGA and GAC) were upregulated during neurogenesis of human NPCs, and their expression pattern positively correlated with the neuronal marker microtubule associated protein 2 (MAP-2; Wang et al., 2014). Most important, studies of cultured human NPCs after siRNA silencing of GLS suggest a critical role of GLS isoforms for proliferation and survival of NPCs (Wang et al., 2014). Furthermore, inhibition of GA activity in cultures of mouse embryonary cortical neurons resulted in impaired neuritogenesis (Velletri et al., 2013), while both mRNA and protein KGA levels strongly increased when cerebellar granule cells differentiate in culture (Thomas et al., 1989). This last study also demonstrated KGA upregulation in parallel with Ca^2+^-dependent glutamate release and formation of neurites, synaptic vesicles and synapses (Thomas et al., 1989). Therefore, it is tempting to speculate that an impairment of KGA in synaptic regions, as expected from the drastic downregulation of KGA shown by KOLPA_1_ mice, would also negatively impact developmental processes of the nervous system.

## LPA and Neuropathic Pain

The perception of noxious stimuli arises from signals originated at the peripheral branches of specialized primary afferents called nociceptors. The cell bodies of these neurons are located at the dorsal root- and trigeminal ganglia and their central processes enter the spinal cord. Nociceptors contact dorsal horn neurons on both projecting and local circuit neurons in specific nociceptive pathways and exert excitatory activity as they use glutamate as their main neurotransmitter. In addition, unmyelinated C-nociceptors also contain neuropeptides such as substance P (SP), which are usually co-released together with glutamate upon tissue injury or persistent stimulation from peripheral nerves, see Millan (1999) for a review. By this means, neuropeptides exert slow excitatory potentials that maintain the initial depolarization elicited by glutamate which results in long-term changes in the responses of dorsal horn neurons through mechanisms resembling those underlying LTP (Sandkühler, 2009). The prolonged changes in excitability are known as central sensitization and help to explain the hyperalgesia (exaggerated response to noxious stimulation) and allodynia (pain in response to innocuous stimuli) that are developed during chronic pain. Sensitization of dorsal horn neurons involves the recruitment of second messenger pathways and activation of protein kinases resembling NMDAR-mediated LTP in brain areas, where LPA was shown to play a key modulatory role. In a similar way, LPA seems critical in the initiation of central sensitization during neuropathic pain, since hyperalgesia and allodynia were absent in LPA_1_ null mice after peripheral nerve damage (Inoue et al., 2004). A single intrathecal injection of LPA in awake mice provoked pain-like behaviors, which were reverted by application of antisense oligonucleotide against LPA_1_ prior to the induction of the experimental neuropathy (Inoue et al., 2004).

**Figure 5:**
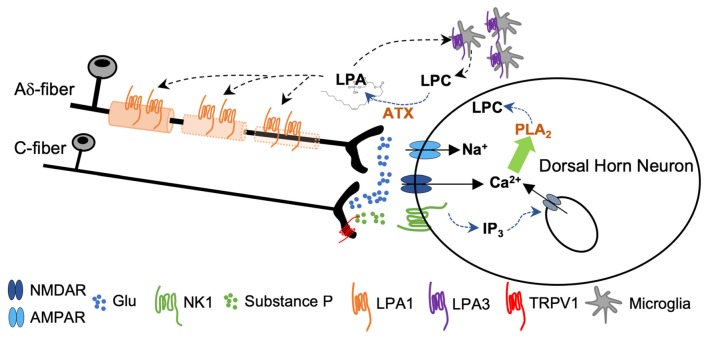
Sustained activity from peripheral nerve as a consequence of nerve damage provokes co-release of glutamate and substance P (SP) from the central terminals of the nociceptors. Activation of NMDA and NK1 receptors provokes a rise in cytosolic Ca^2+^ followed by phospholipase A_2_ (PLA_2_)-induced lisophosphatidilcholine (LPC) synthesis. LPC leaves the cell and is converted to LPA by ATX. Signaling through LPA_1_ initiates a process of demyelination at the central terminals of the primary afferents. Signaling through LPA_3_ constitutes a feed-forward mechanism for LPA formation (Inoue et al., 2004; Fujita et al., 2007; Ma et al., 2010a,b).

Intense activation of primary afferents (by capsaicin, or co-application of SP and NMDA) induced *de novo* LPA synthesis in spinal cord slices by means of an ATX-dependent mechanism (Inoue et al., 2008b). Activity from fast conducting Aδ pain fibers activates primarily AMPAR, causing a rapid depolarization sufficient to release Mg^2+^ block from NMDAR. As a consequence, activity from slow conducting C-nociceptors provokes, in the same post-synaptic neuron, a sustained depolarization *via* NMDA and metabotropic neurokinin 1 receptors for SP (NK1). The intracellular events that follow NK1 and NMDA signaling and lead to LPA accumulation have been largely studied and are summarized in Figure 5. Briefly, signaling *via* NK1 receptors induces sustained excitatory postsynaptic potentials that provokes Ca^2+^ release from the endoplasmic reticulum *via* inositol-1,4,5-triphosphate (IP_3_) sensitive receptors. Meanwhile, PKC-mediated NMDAR phosphorylation potentiates accumulation of intracellular Ca^2+^ and promotes synthesis of LPC by means of phospholipase A_2_ (PLA_2_). LPC leaves the neuron to be converted to LPA by ATX (Inoue et al., 2008a). It has been shown that neuropathic pain behaviors induced by LPC application in the spinal cord were abolished in LPA_1_ and LPA_3_ null mice (Inoue et al., 2004; Ma et al., 2009) and significantly attenuated in ATX^+/−^ heterozygous mice (Inoue et al., 2008b). In addition, *de novo* LPA synthesis was inhibited by PLA_2_ antagonists (Ma et al., 2009, 2010b). LPA causes demyelination in dorsal root fibers when applied *ex vivo* (Fujita et al., 2007); accordingly, nerve-injury induced demyelination was abolished in LPA_1_-null mice and markedly attenuated in ATX^+/−^ heterozygous mice (Inoue et al., 2004; Nagai et al., 2010). In this line, early blockade of LPA_1_ receptors with a specific antagonist Ki-16425 (Ohta et al., 2003) inhibits neuronal damage in the sciatic nerve ligation model and in response to intrathecal LPA injection (Ma et al., 2009). In addition, in a rat model of osteoarthritis, early treatment with Ki-16425 reduced demyelination and nerve damage and attenuated hindlimb pain (McDougall et al., 2017).

LPA-induced demyelination involves at least two different mechanisms. The activation of calcium-dependent protease calpain (*via* G_q/11_) degrades myelin proteins of the Schwamm cells around the dorsal root fibers (Xie et al., 2010). In addition, a sustaining downregulation of myelin proteins is induced by LPA_1_ signaling *via* Gα_12/13_ which activates RhoA and ROCK kinase pathways to silence transcription factors (such as Erg2 and Sox10) involved in myelin gene expression (Fujita et al., 2007).

An intriguing form of neuropathic pain, whose underlying mechanisms are still a matter of debate, is that produced by antineoplastic agents (Park, 2014). Chemotherapy-induced peripheral neuropathy is a common side effect in cancer patients that might require discontinuing the treatment. In mice, it was shown that paclitaxel application, used for the treatment of solid tumors, provoked a marked increase of LPA in the spinal dorsal horn, which was completely abolished in LPA_1_ and LPA_3_-null mice and by intrathecal pre-treatment with NK1 or NMDAR antagonists in WT mice (Uchida et al., 2014).

Hence, nerve insult promotes LPA accumulation by an LTP-like mechanism initiated at NMDA/NK1 receptor of post-synaptic dorsal horn neurons. LPA acts as a retrograde messenger and activates LPA_1_ and LPA_3_ located at the membranes of the Schwann cells and evokes demyelination of the central axon of the primary afferents (Figure 5). At the same time, LPA levels are maintained through *de novo* LPA synthesis promoted by LPA/LPA_3_ interaction at the microglia, since treatment with minocycline reverted both LPA- and nerve damage-induced neuropathic pain and dampened LPA synthesis (Ma et al., 2010b).

Demyelination allows for ectopic relocation of Na_v_ and K_v_ channels along the exposed patches of axonal membrane, altering the normal excitability and prompting abnormal spontaneous firing of action potentials (Roza et al., 2003; Campbell and Meyer, 2006; Bernal et al., 2016; Bernal and Roza, 2018; Roza and Lopez-Garcia, 2008). In addition, loss of myelin could favor electrical coupling between fibers (Meyer et al., 1985; Bernal et al., 2016) and promote aberrant sprouting from demyelinated nodes (Griffin et al., 2010). Nerve trauma, metabolic diseases or viral infection trigger neuropathic pain due to an altered excitability at the peripheral axons of the affected primary afferents; in this context, an involvement of LPA at the site of injury should also be expected. Early experiments showed that LPA injection in the hind-paws of awake mice produced withdrawal reflexes (indicative of pain) which were inhibited by specific blockade of NK1 and pertussis toxin, suggesting the activation of SP-containing nociceptors *via* a signaling mechanism involving PTX sensitive-Gi/o proteins (Renbäck et al., 1999). Furthermore, upregulation of LPA_1_ occurs in the distal nerve stump following nerve injury (Weiner et al., 2001).

Neuropathic pain is a pathological expression of the nociceptive system caused by different etiologies which commonly are present with demyelination. The unequivocal contribution of LPA-signaling in the induction, and possible maintenance (Ueda et al., 2018), of central sensitization points to novel therapeutic strategies for modulation of some neuropathic pain symptoms.

## Conclusions

The bioactive natural lipid LPA induces multiple cellular responses in the CNS as well as in neural cell lines. Many, but not all, of these effects are mediated through interaction with the LPA_1_ receptor, considered the most prevalent receptor type in both embryonic and adult brains of humans and mice. Genetic silencing of LPA_1_ in mice causes altered neurotransmitter homeostasis which were previously related to psychiatric diseases. Behavioral studies with LPA_1_ deficient mice reported deficiencies in spatial memory retention and abnormal use of searching orientation strategies, defective working and reference memory, independently of exploratory and emotional impairments, attributed to hippocampal malfunction. Recent results reinforce the view of LPA as an important mediator of synaptic plasticity. Signaling through LPA_1_ receptor exerts an important impact on KGA expression mainly through a posttranscriptional mechanism, stressing a key relationship between LPA and glutamatergic transmission. Furthermore, the absence of LPA_1_ signaling downregulates expression of active MMP-9 and provokes drastic changes in hippocampal dendritic spines toward an immature phenotype. These changes may provide a molecular basis for the role of LPA in influencing synaptic plasticity associated to cognitive and memory processes. In this line, activation of NMDAR at excitatory synapsis in different areas of the CNS leads to production of LPA which may either potentiate or restrain the synaptic strength by enhancing currents through NMDAR and modulating glutamate release. On the other hand, at inhibitory synapses, NMDAR-induced LPA-synthesis leads to a transient disinhibition on GABAergic neurons. Sustained NMDAR activation in dorsal horn neurons within nociceptive circuits elicits LPA production, which is further sustained by means of a positive-feedback mechanism. Such LPA accumulation initiates demyelination of primary afferents inducing the development of neuropathic pain. Nevertheless, much work is needed to completely address the molecular mechanisms underlying the huge number of key processes affected by this multifaceted bioactive lipid.

## Author Contributions

CR, JC-S, MG-G, AP and JM conceived and wrote the manuscript.

## Conflict of Interest Statement

The authors declare that the research was conducted in the absence of any commercial or financial relationships that could be construed as a potential conflict of interest.
